# Consolidated learning can be susceptible to gradually-developing interference in prolonged motor learning

**DOI:** 10.3389/fncom.2013.00069

**Published:** 2013-05-28

**Authors:** Yuko Yotsumoto, Takeo Watanabe, Li-Hung Chang, Yuka Sasaki

**Affiliations:** ^1^Department of Life Sciences, The University of TokyoTokyo, Japan; ^2^Department of Cognitive, Linguistic, and Psychological Sciences, Brown UniversityProvidence, RI, USA

**Keywords:** motor learning, interference, retrograde interference, finger tapping, consolidation

## Abstract

When multiple items are learned in sequential order, learning for one item tends to be disrupted by subsequently learned items. Such retrograde interference has been studied with paradigms conducted over a relatively short term. Resistance to interference is generally believed to be a measure of learning or consolidation. Here, we used a finger-tapping motor sequence paradigm to examine interference in prolonged motor learning. Three groups of nine subjects participated in training sessions for 16 days, and practiced three different sequences in different orders and combinations. We found that a well-trained motor sequence was subject to a gradual interference when the subsequent learning was paired in a particular order. The results suggest that a well-learned motor memory is still susceptible to interference, and that resistance to interference in one condition does not necessarily imply full, permanent consolidation.

## Introduction

Newly learned information is often transient and thus, inaccessible during later recollection attempts. Thus, learned information can undergo interference by other information or weakened with the passage of time. The process by which information is stabilized and becomes immune to certain types of interfering agents is termed consolidation (Dudai, 2004; Eichenbaum, 2008).

Understanding the functional and neural mechanisms that mediate consolidation is a fundamental issue in neuroscience (Dayan and Cohen, 2011; Censor et al., 2012). Newly learned memories are often unstable, and thus, tend to be disrupted by subsequently learned information. This disruption is called retrograde interference. Retrograde interference can occur in several types of learning, including declarative memory (Alvarez and Squire, 1994), motor learning (Brashers-Krug et al., 1996; Cohen and Robertson, 2011), and perceptual learning (Seitz et al., 2005; Yotsumoto et al., 2009a; Hung and Seitz, 2011). Retrograde interference has attracted considerable attention, as it directly relates to consolidation. In cases where retrograde interference manipulations do not disrupt the performance on acquired memories, it can be assumed that memory consolidation has taken place (Brashers-Krug et al., 1996; Muellbacher et al., 2002; Walker et al., 2003a). Retrograde interference is typically evaluated with an A-B-A paradigm (Walker et al., 2003a; Krakauer et al., 2005). In this paradigm, task A is learned before task B, and repeated after learning task B. If task A performance is disrupted after learning task B, then task B is considered to have caused retrograde interference. If learning task B does not disrupt task A performance, then task A is considered to be “consolidated.”

Previous motor consolidation studies largely focused on interference generated between motor skills acquired over a short time interval that lasted up to a few days (Brashers-Krug et al., 1996; Muellbacher et al., 2002; Walker et al., 2003a). In those studies, using A-B-A paradigms, the first learning of task A and task B was conducted within the same day, and task A was subsequently retested on the next day. The interval between task A and task B on the first day was manipulated to examine the temporal characteristics of consolidation and retrograde interference. When task A and task B were temporally apart for more than 4–6 h, task B did not disrupt the learning of task A, hence task A was considered as consolidated (Brashers-Krug et al., 1996; Walker et al., 2003a). Nevertheless, immunity to interference over such a short time period may not necessarily indicate immunity to other forms of interference. In fact, several studies have shown that memory might never become permanently immune to disruption (Misanin et al., 1968; Nadel and Land, 2000; Nader et al., 2000). Using fear conditioning procedures and electroconvulsive shock to hippocampus cells in mice, Nader et al. (2000) demonstrated that consolidated memories can be reactivated by a conditioned stimulus and become labile to disruption again. The observation that reactivation can render memories labile to modifications is termed reconsolidation (Przybyslawski and Sara, 1997).

Here, we show that human motor memories can also experience interference after it was considered consolidated. We observed that a well-trained motor skill acquired with long-term training was resistant to interference in some conditions, but was subject to strong interference when an interfering motor task was introduced together with the well-trained motor task, and the interfering task preceded the well-trained task. Our results indicate that motor memory might also follow the rules of consolidation and reconsolidation when memory reactivation is applied after prolonged periods of motor learning.

## Methods

### Subjects

A total of 27 right-handed subjects (13 females, 14 males) participated in the study. Subject ages ranged from 18 to 34 years (mean, 22.0 years; standard deviation, 3.8 years). None of the subjects played a musical instrument. All subjects gave informed written consent. The Institutional Review Board of Massachusetts General Hospital approved the study.

### Procedure

Subjects trained to perform a finger-tapping task (Karni et al., 1995, 1998) in 16 training sessions. Subjects participated in at least three training sessions per week, which took place on separate days with a 1- to 2-day interval between sessions. Thus, the total training period spanned 18–26 days (mean = 22.63 ± 2.43 days). Subjects trained with a single sequence on Days 1–8, and then trained with two sequences on Days 9–16. For Days 1–8, training sessions were ~12 min in duration, and consisted of twelve 30-s blocks, with 30-s resting periods between blocks. For Days 9–16, training sessions were ~24 min in duration, and consisted of twelve 30-s blocks for each sequence (24 blocks in total), with 30-s resting periods between blocks.

**Figure 1:**
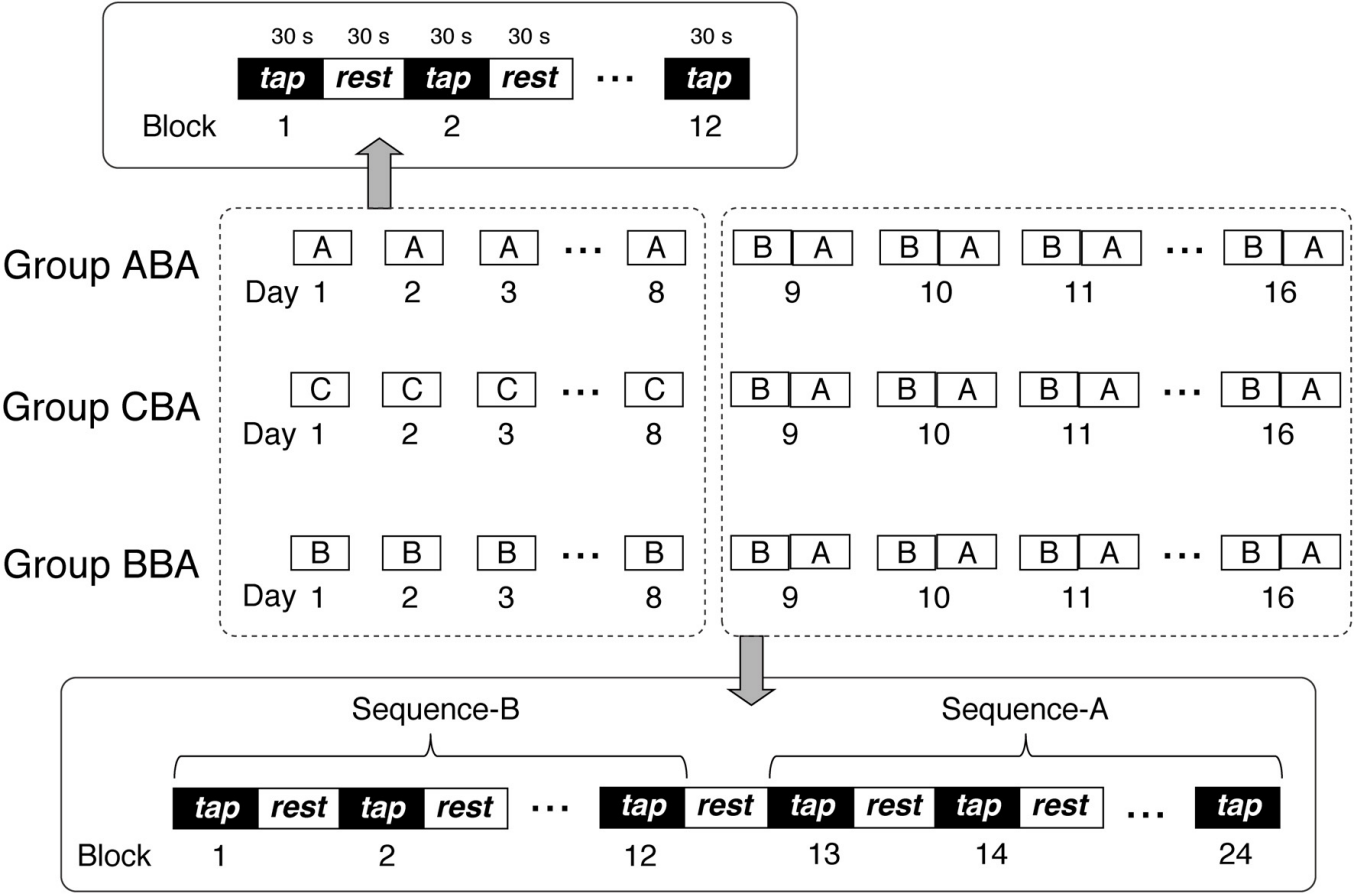
**Schematic representation of the experimental procedure**.

Subjects were divided randomly into three groups: Group ABA, Group CBA, and Group BBA. Figure 1 presents a schematic representation of the training sessions for the three groups. During Days 1–8, each group performed a different training sequence. Group ABA trained with sequence A, Group BBA trained with sequence B, and Group CBA trained with sequence C. The training sequences performed on Days 9–16 were identical for all groups, which included sequence B for the first 12 blocks followed by sequence A for the second 12 blocks.

Subjects were instructed to tap four keys on a keypad (Nostromo SpeedPad, BELKIN) according to the instructed sequence, using the fingers of their non-dominant hand. Each of the four keys was assigned a number between 1 and 4, with 1 corresponding to the little finger, 2 to the ring finger, 3 to the middle finger, and 4 to the index finger. Subjects were instructed to tap the keys as quickly and accurately as possible for 30 s. A sequence was five key presses arranged in one of the three following orders: sequence A [4-3-1-2-4], sequence B [4-2-3-1-4], and sequence C [4-1-3-2-4]. The assignment was counter balanced across subjects; therefore, sequence A could require different tapping sequences between subjects.

All experimental sessions were conducted in a laboratory. The instructed tapping sequence was displayed on the center of a computer screen throughout the task to eliminate the requirement for working memory. On Days 9–16, when two sequences were performed, subjects were reminded twice about the sequence change before the training session and immediately before the second sequence began. Both the keypad and the tapping hand were hidden from the subject's view during the task.

### Data analysis

The number of correctly tapped sequences was averaged across 12 blocks for each subject. The resulting mean was used as an index of motor learning.

## Results

### Group ABA

**Figure 2:**
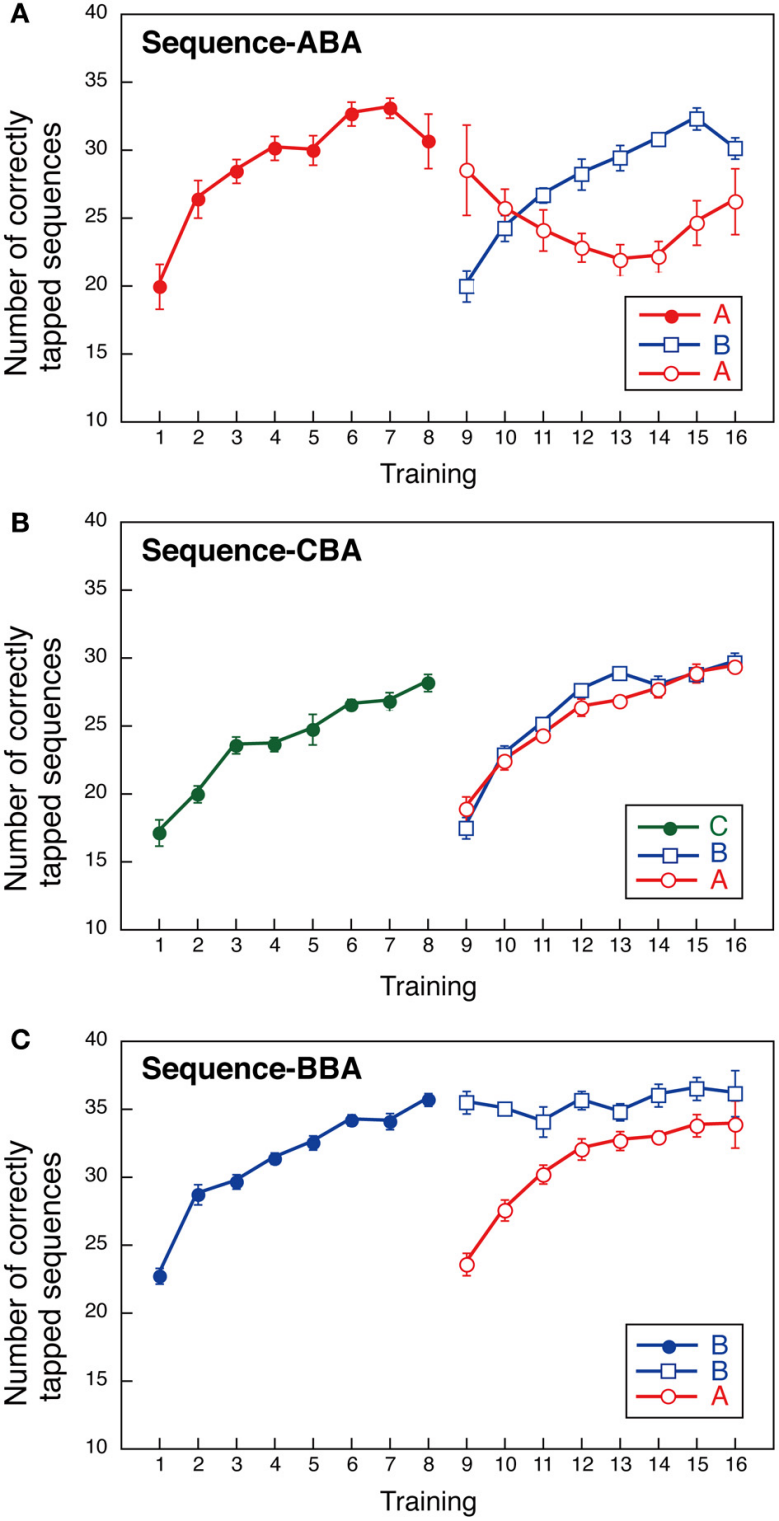
**Performance during a finger-tapping motor sequence task for three training sequences (three groups).** The mean number of correctly typed responses as a function of the training day. In the first part of the training session (Days 1–8), a single sequence was trained; two sequences (sequences **B** and **A**) were practiced in all groups in the second training period (Days 9–16). **(A)** Group ABA; **(B)** Group CBA; **(C)** Group BBA.

Group ABA first practiced sequence A, then sequence B, followed by sequence A. The number of correctly tapped sequences for sequence A increased gradually during training and plateaued by Day 8 (Figure 2A). Notably, sequence B performance also improved gradually at a rate similar to that observed for sequence A. In contrast, performance in the second training instance for sequence A was diminished when compared to the first sequence A training session. That is, we found interference for sequence A after sequence B was learned. Sequence A performance improved again once sequence B performance plateaued (see Days 14–16 in Figure 2A). We conducted a Two-Way ANOVA (day vs. sequence) with repeated measures to compare the two instances of sequence A performance, and between sequence A and sequence B performance. This revealed significant effects for “day” [*F*_(7, 56)_ = 7.654, *p* < 0.001], and a significant interaction between “day” and “sequence” [*F*_(14, 112)_ = 5.112, *p* < 0.001]. No significant effect was observed for sequence [*F*_(2, 16)_ = 1.470, NS]. These findings support our hypothesis that learning sequence B introduces interference on the previously learned sequence A.

### Group CBA

Group CBA first practiced sequence C, followed by sequence B, and then sequence A. For Group CBA, the number of correctly tapped sequences for sequence C increased gradually over training and plateaued by Day 8. During Days 9–16, the number of correctly tapped sequences also increased gradually, both for sequences B and A (Figure 2B). Although, sequence B was the second sequence for all groups, Group CBA did not show signs of interference across sequences. A Two-Way ANOVA (day vs. sequence) with repeated measures performed with Group CBA data revealed a significant main effect for day [*F*_(7, 56)_ = 68.657, *p* < 0.001]. The sequence [*F*_(2, 16)_ = 2.800, NS] and the interaction between day and sequence, were not significant [*F*_(14, 112)_ = 1.342, NS].

### Group BBA

Group BBA first practiced sequence B, repeated sequence B, and then performed sequence A. The sequence presented in the second part of training was identical to that for Groups ABA and CBA. If the well-trained sequence was subject to interference, then sequence B performance during the second training period should worsen over several days. Our results, however, revealed sequence B performance improvement was maintained throughout the second training period (Figure 2C). In addition, sequence A performance improved across days. That is, no interference was observed between sequences A and B. A Two-Way ANOVA (day vs. sequence) with repeated measures showed significant main effects for day [*F*_(7, 56)_ = 17.838, *p* < 0.001] and sequence [*F*_(2, 16)_ = 16.578, *p* < 0.001], and a significant interaction between day and sequence [*F*_(14, 112)_ = 8.249, *p* < 0.001]. The significant main effect for the sequence and the significant interaction between day and sequence reflect that the sequence B performance in the second period sustained while the sequence A performance improved.

### Relative performance improvement

Among the three experimental groups, performance decline was observed only in Group ABA. To assure that such decline did not result from differences in motor learning skills, we compared the relative performance improvements during the first 8 training days. Had the improvement in the first 8 days differed across groups, the differences observed in the second training period could have been attributed to other factors, such as individual differences in motor skills.

**Figure 3:**
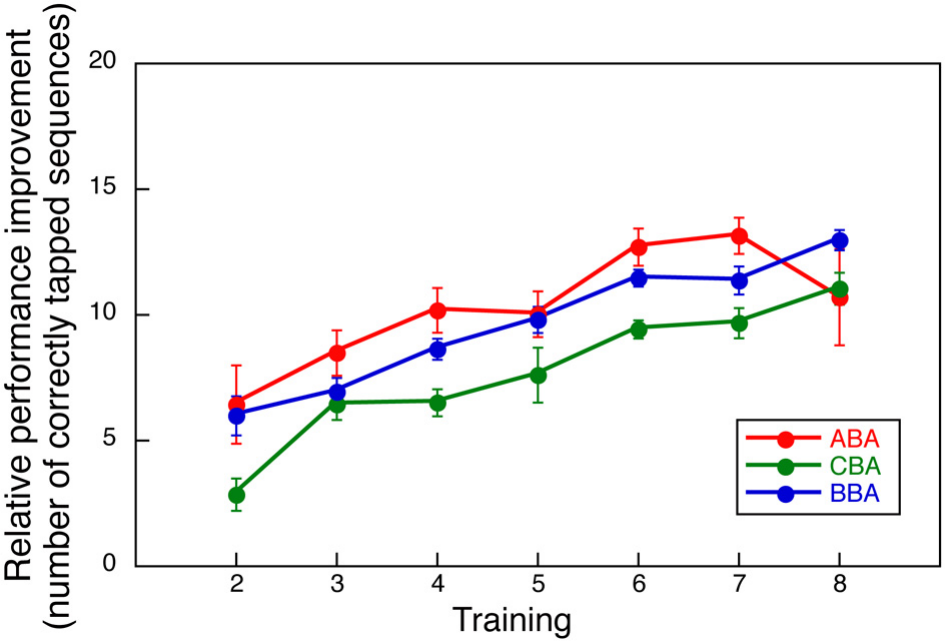
**Relative performance improvement compared to the initial day performance by group**.

The relative improvement was computed by subtracting the number of correctly tapped responses on Day 1 from those observed on Days 2 through 8. Figure 3 shows the averaged relative performance improvement for all three groups in the first part of the training. A Two-Way ANOVA with repeated measures, with group and days as factors, indicated no significant effect for group [*F*_(2, 24)_ = 1.048, NS] or interaction between day and group [*F*_(12, 144)_ = 0.954, NS], though, a significant main effect was observed for day [*F*_(6, 144)_ = 21.783, *p* < 0.0001]. This measure indicates that overall learning performance for all groups was comparable.

### Within-session learning

**Figure 4:**
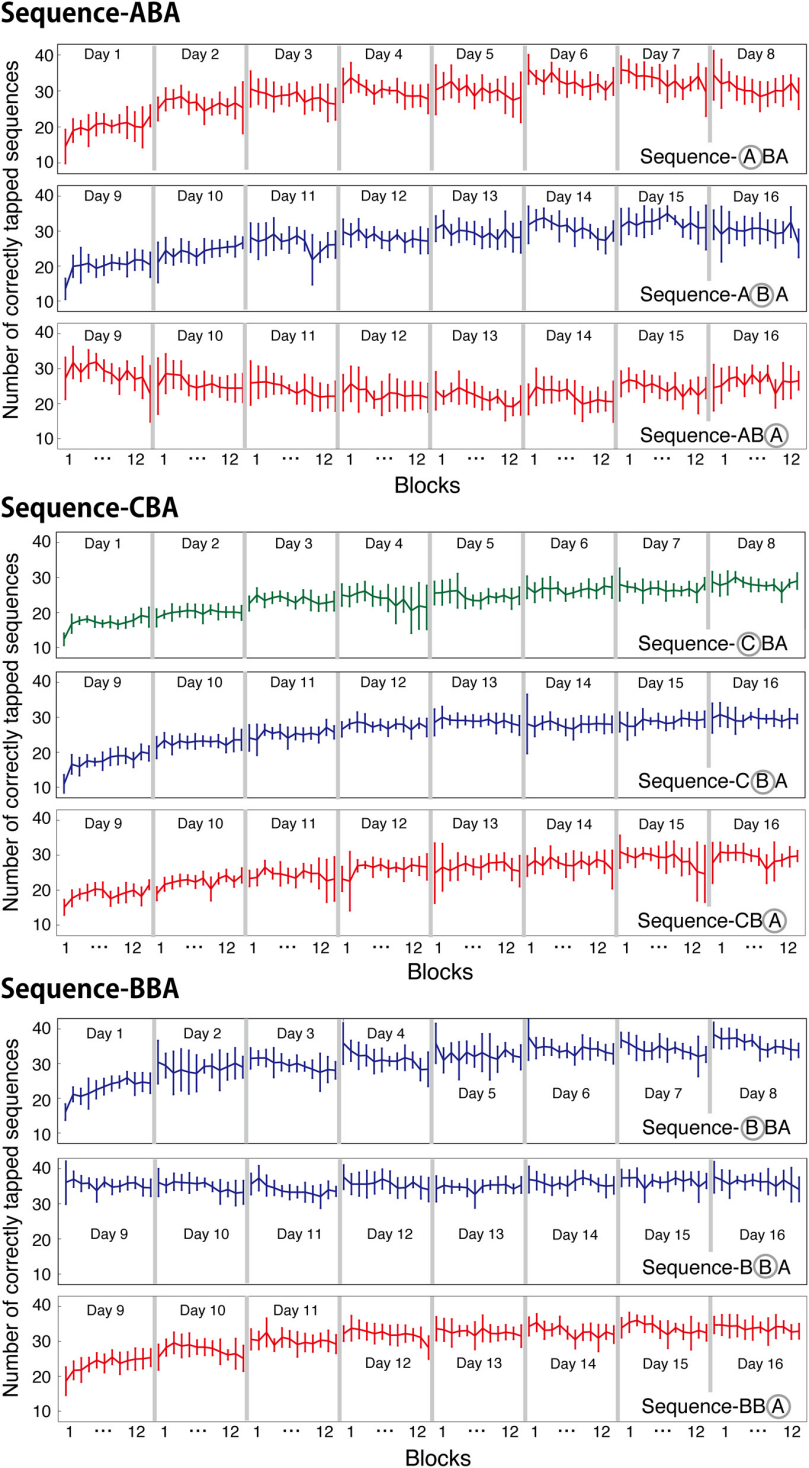
**Averaged number of correctly tapped sequences (±s.e.m.) plotted for each block and for each session.** For each group, the mean numbers of correctly tapped sequences are plotted for each learned sequence.

The number of correctly tapped sequences shown in the previous figures were averaged across 12 blocks within a session. In the present motor task, one session consisted of twelve 30-s blocks. It is well-known that motor learning performance improves not only across sessions but also within a session, especially during the initial learning session (Karni et al., 1998). To examine within-session learning, we counted the number of correctly tapped sequences for each block and for each session, and plotted the counts in Figure 4. For all three groups, the newly introduced sequences exhibited within-session improvement during the initial training session (Day 1 and 9). For other training sessions (days), the task performance was consistent across the 12 blocks.

### Across-session learning vs. within-session learning

**Figure 5:**
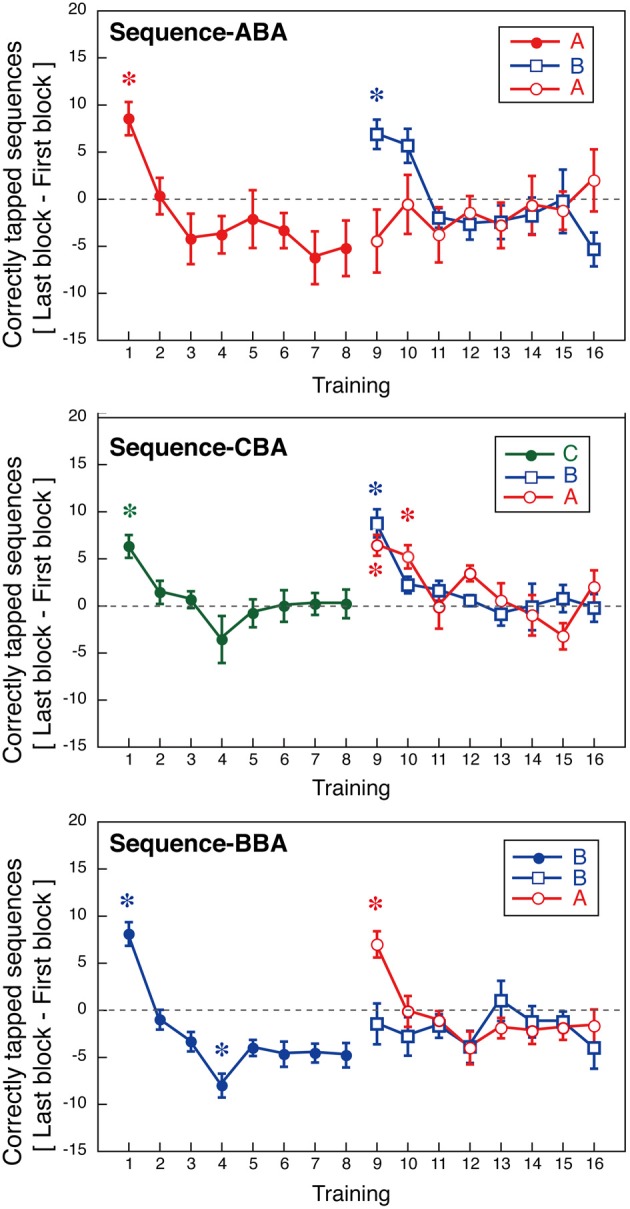
**Within-session learning as indicated by an increase in the number of correctly tapped sequences within a session (±s.e.m.).** Asterisks indicate sessions with significant deviation from zero (*p* < 0.05, Bonferroni correction).

To further, examine the nature of within-session learning, we calculated the difference in the number of correctly tapped sequences between the first and last blocks of each session. These values are presented in Figure 5. These values are an index of within-session learning, with positive values indicating within-session learning, and negative values indicating within-session deterioration.

For Days 1–8, within-session learning was evident only in the first or second sessions. For all three groups, the first training session significantly differed from zero [ABA: *t*_(8)_ = 4.09, *p* < 0.05; CBA: *t*_(8)_ = 8.81, *p* < 0.05; BBA: *t*_(8)_ = 5.49, *p* = 0.05; one-sample *t*-tests, Bonferroni correction]. After, the second training session, subjects showed no within-session learning until a new tapping sequence was introduced on Day 9. In Group BBA, subjects showed a negative value for the within-session learning on Day 4, which might be explained by fatigue [*t*_(8)_ = −4.9, *p* < 0.05, Bonferroni correction].

The across-session learning (Figure 2) was calculated by averaging the numbers of correctly tapped sequences across the 12 blocks. In contrast, within-session learning (Figure 5) was calculated from the difference between the first and last blocks of a session. These two types of learning showed distinct pattern changes as the training progressed. While the average number of correctly tapped sequences across 12 blocks continually increased from the first to the eighth session, within-session learning was evident only in the first training session. This difference implies there are two types of motor learning, one characterized by gradual training increases across days, and one characterized by quick changes within a session that disappears in relatively early training phases.

For the second half of the learning phase, Group ABA showed interference in across-session learning, and the pattern of within-session learning for sequence B differed from that for sequence A. A Two-Way ANOVA (day vs. sequence) with repeated measures confirmed a significant interaction between days and the trained sequence [*F*_(7, 56)_ = 2.56, *p* = 0.023] during the same training period. For Group ABA, within-session learning for sequence A during Days 9–16 was insignificant (*p* > 0.25 for all sessions), indicating no signs of interference. The difference between the across-session and the within-session learning suggests that interference from learning other sequences primarily affected long-term across-session learning.

### Correlations between interference and individual performance

In the previous analyses, we describe interference by pooling the number of correctly tapped sequences across subjects. One might wonder if any individual differences might affect such interference effects. For example, an individual who performs well in their first training session might have some resistance to interference, thus, causing less interference in the second part of the training. To examine the effects of individual performance on interference, we calculated the performance in the first training session, and the extent of interference for all subjects in Group ABA. We then examined correlations between these measures.

Performance in the first training session was quantified by the number of correctly tapped sequences (A). To determine the extent of interference, we first selected for each subject the session in which the largest number of correct sequence A trials were performed during Days 1–8. In addition, we also selected for each subject the session with the fewest completed correct sequence A trials during Days 9–16. Assuming the session with the worst performance was the session with largest interference, we subtracted the worst performance during Days 9–16 from the best performance during Days 1–8. This value indicates how much deterioration was caused in sequence A tapping by introducing sequence B.

**Figure 6:**
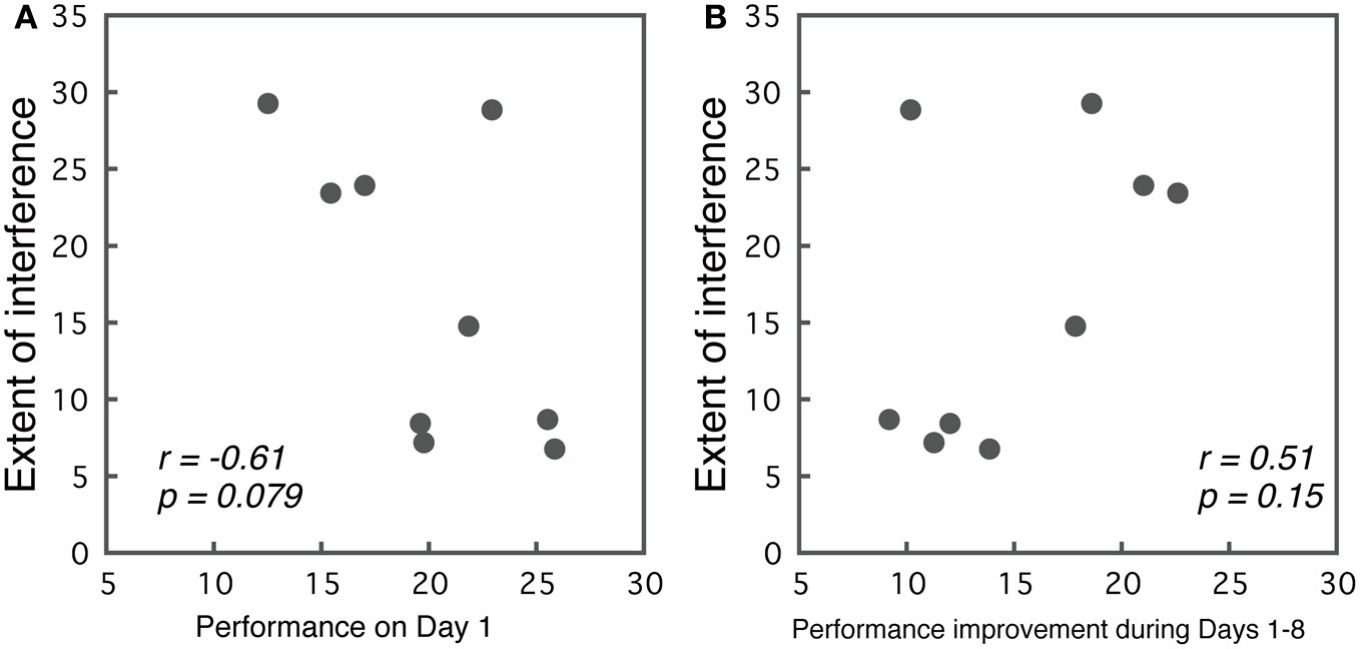
**(A)** Correlations between the initial performance on Day 1 and the extent of interference. **(B)** Correlations between the performance improvement during the first part of the training, and the extent of interference.

Correlations between initial performance (Day 1) and the extent of interference (best performance in the first part—worst performance in the second part) are presented in Figure 6A. We observed a trend indicating that better performance on Day 1 predicted less interference (*r* = −0.61, *p* = 0.079). Although, the results were not significant, they implied that the initial performance level may contribute to the occurrence of interference.

While, we did not find significant effects between initial performance and interference, the extent of learning might relate to the extent of interference. To examine this possibility, we calculated correlations between the performance improvement during Days 1–8, and interference in Group ABA. For the Days 1–8, we selected for each subject the session with the best performance, and calculated the difference between the best performance and their performance on Day 1. This value indicates the extent of improvement made in the first part of the training. Correlations between the amount of learning and the amount of interference are presented in Figure 6B. As the figure indicates, a weak trend was observed in that subjects who showed larger improvements during Days 1–8 also showed larger interference, though, this was insignificant (*r* = 0.513, *p* = 0.15).

## Discussion

Our present findings indicate that performance on a well-trained motor skill is subject to interference when learning of a new sequence is inserted before performing the well-trained skill. Such interference was not observed if the order of learning is reversed for the new and the well-trained skills.

Can diminished performance of sequence A in the second part of training for Group ABA be attributed to a factor other than interference from sequence B? First, it is unlikely that diminished performance in sequence A was attributed to a limited capacity of motor memories, since improved performance on both sequences A and B was observed in the second part of training in Groups CBA and BBA. Second, it is unlikely that poorer performance on sequence A for Group ABA was due simply to switching from one sequence to another (Imamizu et al., 2007). If there was a cost for switching sequences within a session for Group ABA, we should have observed decline in the earlier blocks of the interfered sessions. However, the block-wise analysis shown in Figure 4, and the within-session improvement shown in Figure 5, both indicate performance was consistently interfered within a session. Third, it is unlikely that extended practice of the same sequence decreased subject motivation for typing the correct responses, which would have led to poorer performance on the sequence performed last. We believe this because improved performance on sequence B did not disappear in Group BBA in the second part of training. If prolonged practice bored the subjects, then performance on sequence B would have been lower in the second part of training in Group BBA. Fourth, it could be argued that subjects in Group ABA showed diminished performance because of poor learning. This, however, is unlikely. While, average performance on Day 1 was slightly different across the three groups (Group CBA was the lowest in initial performance), average improvement over the 8 daily sessions in the first part of training was almost identical across groups (see the slope for Days 1–8 for the three groups in Figure 2 and also Figure 3). Finally, one may argue that retrieval of presumably consolidated information alone makes the memory once again susceptible for interference, as was shown in a previous study (Walker et al., 2003a). Nevertheless, this may not be the case. If the retrieval of memory alone made the memory fragile, then the performance of sequence B would have been worsened in the second part of the training for Group BBA.

While, no interference was observed for learning sequence A or B in Group CBA, results from previous studies (Brashers-Krug et al., 1996; Muellbacher et al., 2002; Walker et al., 2003a) indicate that retrograde and/or anterograde interference between sequences A and B was more likely when the training of the two new sequences was temporally close (within a few days). Those studies, however, did not include a condition that consisted of learning a different sequence as employed in the current study in Group CBA. Perhaps enhanced general ability (Krakauer et al., 2005) in motor skill learning that resulted from first learning C in the training for Group CBA made an efficient neural representation (Yotsumoto et al., 2008) for a new motor skill, leading to the successful acquisition of two new (sequences B and A) motor skills.

Neural representation of consolidated motor skill is different from that of acquisition (Brashers-Krug et al., 1996; Shadmehr and Holcomb, 1997; Muellbacher et al., 2002; Dayan and Cohen, 2011). In addition, different neural systems for consolidation are proposed for two types of motor skill learning. These two systems are dynamic motor adaptation learning and motor sequence learning (Doyon et al., 2003; Doyon and Benali, 2005). For both dynamic motor adaptation learning and motor sequence learning, the condition under which retrograde interference occurs has been identified as successive acquisition of two motor skills presented within a short temporal interval between the first and second skill (Muellbacher et al., 2002; Doyon and Benali, 2005; Krakauer et al., 2005; Krakauer and Shadmehr, 2006). The present study found that interference occurred for retrieval of well-trained motor sequence learning: In Group ABA, sequence A was presumably reinstated by the learning of sequence B, thus, rendering it labile to interference. Because the learning of sequence B interfered with the previously learned sequence A, the interference should be construed as retrograde. Whether our findings can be applied to dynamic motor learning, or whether they apply to motor sequence learning, remains open to investigation.

Although, the neural mechanisms for prolonged motor learning have yet to be revealed, prolonged visual perceptual learning has been reported to induce transient changes in neural activity. Using a texture discrimination task (Karni and Sagi, 1991), Yotsumoto et al. (2008) examined dynamics of brain activation in visual cortex across 14 training sessions conducted on different days across 3–4 weeks. While, the behavioral performance gradually increased for about a week and reached a plateau for rest of the training period, the BOLD signal changes in primary visual cortex (V1) exhibited a different pattern: the task-relevant BOLD signal in V1 increased for about 10 days, then returned to its baseline toward the end of the training period. This indicates that even when behavioral performance seems similarly sustained, the underlying neural mechanisms might change as training progresses. Although, visual perceptual learning in a texture discrimination task involves different cortical areas from motor learning with finger tapping paradigm, the tasks are analogous in terms of consolidation and interference. The learned feature becomes consolidated during sleep after the initial learning (Mednick et al., 2002; Walker et al., 2002, 2003b; Yotsumoto et al., 2009b), and becomes resistant to the retrograde interference (Karni and Sagi, 1991; Walker et al., 2003a; Yotsumoto et al., 2009a). In the present study, 16 training sessions were conducted in 18–26 days. It is reasonable to hypothesize that prolonged motor learning in the present study induced transient changes, making neural states differ between the earlier phase and the later phase of the learning. In the present study, the repeated learning sessions during the first part of training could have enhanced neural plasticity and modified the neural state from that of the motor learning tested with short intervals. The interference observed in our prolonged learning could be attributed to the neural state of the consolidated motor memory. While the motor memory has been reported to become resistant to retrograde interference within a day (Brashers-Krug et al., 1996; Walker et al., 2003a), the present study indicated that the neural state of the motor memory could become vulnerable again to the retrograde interference after the repeated learning. Such vulnerability to the interference may lead to reactivation and reconsolidation of the motor memory. It is implied that the interference in prolonged learning may stem from the later stage of neural consolidation, whereas, the interference in short interval motor learning may stem from the earlier stage of neural consolidation. Yet, it is unclear why the order of the two sequences learned in the second part of training was critical for interference, and how the prolonged motor learning is neurally consolidated. Future studies are needed to clarify the underlying neural correlates of full consolidation in motor skill learning.

To conclude, a well-learned motor memory can be susceptible to retrograde interference, and resistance to interference in the earlier stages of learning does not necessarily indicate full, permanent consolidation for that skill.

## Author contributions

Yuko Yotsumoto, Takeo Watanabe, and Yuka Sasaki designed the experiment. Yuko Yotsumoto and Li-Hung Chang collected data. Yuko Yotsumoto and Yuka Sasaki analyzed data. Yuko Yotsumoto, Takeo Watanabe, and Yuka Sasaki wrote the paper.

### Conflict of interest statement

The authors declare that the research was conducted in the absence of any commercial or financial relationships that could be construed as a potential conflict of interest.
